# Smart Speakers as an Environmental Control Unit for Severe Motor Dependence: The Case of a Young Adult with Duchenne Muscular Dystrophy

**DOI:** 10.3390/ijerph21060778

**Published:** 2024-06-14

**Authors:** Rafael Tavares, Andreia Inácio, Helena Sousa, Jaime Ribeiro

**Affiliations:** 1 Polytechnic Institute of Porto, 4200-072 Porto, Portugal; rafael.tavares@ipleiria.pt; 2 Instituto Politécnico de Leiria, 2411-901 Leiria, Portugal; 3 Centro de Investigação em Reabilitação (CIR), Polytechnic Institute of Porto, 4200-072 Porto, Portugal; 4 Assistive Technology and Occupational Performance Laboratory (aTOPlab), Center for Innovative Care and Health Technology (ciTechCare), Instituto Politécnico de Leiria, 2414-016 Leiria, Portugal

**Keywords:** DMD, assisted living, Internet of Things, caregiver burden, participation

## Abstract

Duchenne muscular dystrophy (DMD) is a disease that primarily affects males and causes a gradual loss of muscle strength. This results in a deterioration of motor skills and functional mobility, which can impact the performance of various occupations. Individuals with DMD often rely heavily on caregivers to assist with daily activities, which can lead to caregiver burden. A case study was conducted to explore and describe potential variations in the performance of a young adult diagnosed with DMD and his caregivers resulting from the integration of smart speakers (SS)-controlled Internet of Things (IoT) devices in the home environment. The study also examined the potential of SS as an environment control unit (ECU) and analysed variations in caregiver burden. Smart devices and SS were installed in the most frequently used spaces, namely, the bedroom and living room. The study employed WebQDA software to perform content analysis and Microsoft Excel to calculate the scores of the structured instruments. The implementation of the IoT-assisted environment compensated for previously physical tasks, resulting in a slight increase in independent performance and reduced demands on caregivers.

## 1. Introduction

Duchenne muscular dystrophy (DMD) is an X-chromosome-linked, male-predominant genetic disease caused by the absence of functional dystrophin, a structural protein in the cytoskeleton of myofibrils [1,2].

Epidemiological data suggest a global prevalence of manifest disease of approximately 7.1 cases per 100,000 males and 2.8 cases per 100,000 in the general population [3].

The reduction in dystrophin causes damage to the myofibrils, reducing strength, stability, and functionality. This results in chronic muscle inflammation, progressive fibrosis, and muscle stem cell dysfunction [1]. Progressive muscle deterioration has several systemic consequences, including delayed motor development, with the average age of gait acquisition being 18 months, with records of onset at 24 months, subsequent loss of ambulation, reduced respiratory capacity, gastrointestinal dysregulation, and cardiomyopathies, as well as potential changes in psychosocial behaviour [4].

Gradual muscle loss and the resulting deterioration in motor skills for functional mobility are associated with changes in the performance of different occupations, even leading to situations where activities cannot be performed without assistance from others [5].

Individuals with DMD often rely on family support and are highly dependent on caregivers to carry out their daily routine. Caregivers, in turn, tend to be overburdened [6,7]. The proposal by Zarit et al. [8] defines burden as the feeling of suffering in terms of physical or mental health, social life, and financial status on the part of the caregiver as a result of caring for the family member. This definition is widely accepted in the literature [9,10].

DMD can have a significant impact on various aspects of a carer’s life, including quality of life, sleep, family relationships, mental health, pain, stress, sexual function, self-esteem, and work productivity [10]. Time spent on informal care and household expenses are two important risk factors associated with these adverse outcomes [7].

Therefore, enhancing the independent participation of individuals with DMD in various occupational areas is necessary. One possible approach is to compensate by alternative means of controlling surrounding environmental factors and assistive technology comes up as an enabling factor. According to the WHO-UNICEF global report, assistive technology is “the application of organized knowledge and skills related to assistive products, including systems and services” p. 6 [11]. This and other interventions related to assistive technologies (AT) are relevant both in ambulatory and non-ambulatory phases, particularly when the decrease in upper limb strength affects reaching, fine motor skills, and activities of daily living [12,13,14]. Subsequently, contemporary assistive technology aims to replace physical movement and reduce fatigue, thereby improving functional efficiency and independence in the task [14].

Smart home systems are modern solutions that use wireless networks to allow connected devices to communicate with each other based on commands obtained from the user interface or pre-defined automations [15]. This vision of a connection between any physical or virtual thing—i.e., object or software—that benefits from an Internet connection is taken up in the concept of the Internet of Things (IoT) [16].

The commercialisation of smart home devices presents opportunities for adapting environments with personalised interfaces. Smart speakers, such as Google Nest or Alexa, can be used as environmental control units (ECUs) [17]. They enable voice activation, eliminating the need to move limbs to activate them [17,18]. Individuals with significant mobility impairments who still possess the ability to articulate speech have the opportunity to operate multimedia devices for leisure, work, or educational purposes, as well as to adjust and manage their environment in terms of lighting, temperature, and safety [17].

The study of environmental adaptation through the introduction of IoT devices controlled by smart speakers (SS) is an emerging area in the literature, especially in observations with the elderly population to promote ageing in place [19,20]. In disabled populations, compensatory strategies are sought for sensory deficits, mainly visual, mobility, cognitive, and emotional deficits [21]. To the authors’ knowledge, this is the first study explicitly exploring environmental adaptation using IoT devices controlled by SS in DMD. Therefore, there is a need to provide evidence in this area. To this end, the following research question was formulated: “How does the use of smart speakers as an environmental control device affect the occupational performance of a person with Duchenne muscular dystrophy and their caregivers?”.

## 2. Materials and Methods

This study takes a qualitative approach, although it employs quantitative measures derived from structured instruments for the purpose of further characterisation. It was designed with exploratory and descriptive aims to analyse an environmental intervention in a real, natural and specific residential context [22]. It is a case study that aims to describe possible variations in the participation and performance of a young adult diagnosed with DMD by integrating IoT devices controlled by SS into his home environment. It will also consider the potential of SS as an ECU and analyse variations in carer burden.

To achieve these aims, the case study research is considered the best methodological design to better understand this unexplored context. While randomised clinical trials reduce many threats to internal validity, the mechanisms of effect remain opaque, particularly when the causal pathways between “intervention” and “effect” are long and potentially non-linear. Case study research plays a key role in providing detailed observational evidence for causal claims [22,23].

### 2.1. Participants

One individual was intentionally selected to meet the following inclusion criteria:

- Diagnosis of DMD;
- Aged between 18 and 23 years;
- Main activities performed in the home environment;
- Possession of pre-existing resources in the home—specifically, an Internet network and adaptable multimedia devices that are not part of the IoT concept.

Individuals with continuous use of an orally applied lung ventilator were excluded due to speech production and comprehension difficulties.

Cohabitees, the participant’s father and mother, who take on the role of carers, were included in the study to increase the diversity and depth of the data generated, obtain different perspectives, and help triangulate the data.

S.T. is a 22-year-old male who has been diagnosed with Duchenne muscular dystrophy. Currently, he is not participating in any training or professional activities. He completed a higher professional technical course (TeSP) in the field of environment, heritage, and sustainable tourism. The course was initially taught in person but was completed through distance learning due to the COVID-19 pandemic. The primary carer, the mother, stopped working to stay at home, while the father established his workplace on the house’s premises. As a result, both parents have become full-time carers.

The following section outlines S.T.’s health and functionality information, categorised according to the International Classification of Functioning, Disability, and Health (ICF) [24]. The codes selected are based on the ICF core set for neuromuscular disorders validated by Bos et al. [25], albeit with deliberate variations, including the addition of structural codes (s) and the conversion of activity (a) and participation (p) codes to performance (d) codes.

#### Functional Profile of the Participant

S.T. experiences reduced energy levels due to the hypotonic state associated with DMD (b1300.3, b740.4) (the explanation of the use of ICF qualifiers is described in Supplementary Files Tables S1 and S2).

There are no reported cognitive difficulties based on anamnesis and direct observation.

S.T. is able to focus on a specific stimulus, except when experiencing overlapping pain sensations from sitting, and lacks the capacity for voluntary defensive postural readjustment (b140.1, b280.3, b760.4, d415.4). The individual has difficulty ideating for future goals (b144.0, b160.1) but can store and recall information and organise his thoughts. While the family routine contributes to a structured sleep cycle, there are challenges in independently changing positions (b134.2, d410.4).

There is no record of psychiatric changes, although the possibility of unmonitored emotional changes cannot be excluded (b152.8).

Despite the common record of visual changes in muscular dystrophies, S.T. retains functional vision without needing compensation with glasses (b210.0, e1251 + 0).

S.T. is capable of accurately articulating the phonemes required for verbal communication, resulting in clear and easily perceptible speech sounds (b320.0). However, dysfunction of the respiratory muscles can hinder adequate projection, volume, and nasality, although it does not prevent the production of explicit verbal messages that can be used to maintain a conversation (b310.1, b455.3, s430.278, d330.1, d350.1). The individual uses a ventilator with a nasal mask at night (e115 + 3). The individual utilises various means of communication, such as sending messages via SMS, email, or chat. However, third parties are required to prepare the layout and activate the necessary devices for this purpose (d360.2).

The aforementioned hypotonia can be classified as severe, according to Campbell’s hypotonia classification scale (b735.4) [26]. Low basal muscle tension is associated with a reduction in the force produced by muscle contraction and the ability to sustain contractions, resulting in an inability to respond involuntarily to balance-related stimuli (b730.4, b740.4, b755.4). There is no abnormal involuntary movement (B765.0). It is in the non-ambulatory stage and manifests sensations of rigidity, tension, contracture, and muscular hardness (b770.4, b780.3, d450.4).

**Figure 1:**
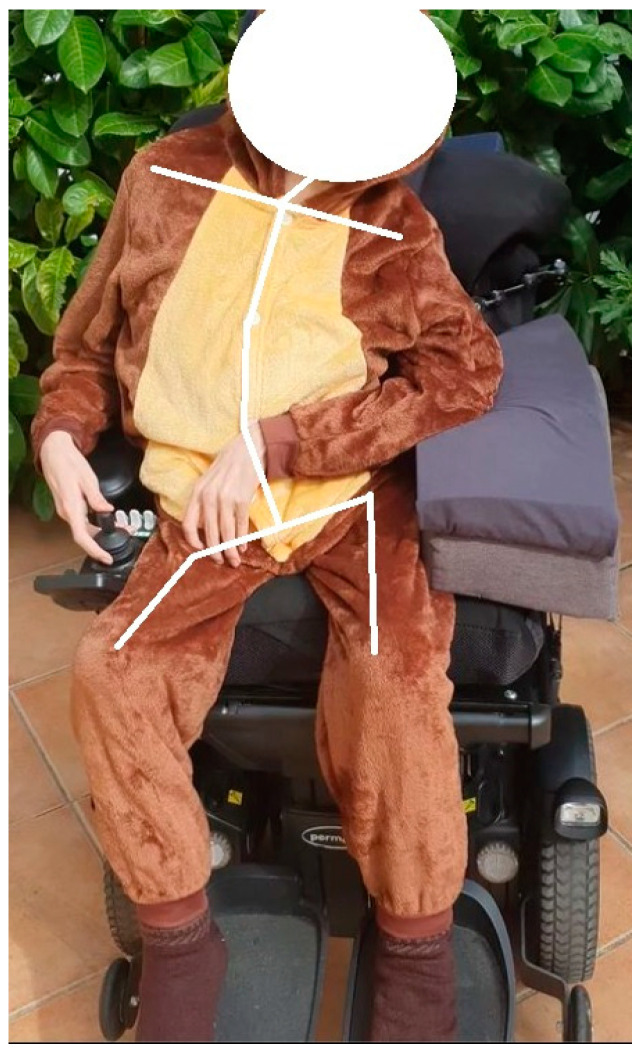
S.T. postural alignment.

Figure 1 shows several permanent structural alterations. The pelvis is in anteversion with right rotation and left obliquity (s740.360). This anteversion is associated with marked lumbar hyperlordosis, high-angle scoliosis, and compensatory contralateral thoracic scoliosis. These conditions result from postural collapse due to muscular inability to support the mass of the upper trunk, upper limbs, and head (s760.460, s770.470). The participant presents misaligned shoulders with a noticeable elevation to the right and shortening of the flexors in the upper limbs, which hinders passive mobilisation throughout the entire range of motion (s720.363, s730.373). Additionally, there is a marked left tilt in the neck, compensated at the head level to align the field of vision (s710.362). Similarly, the lower limbs also exhibit significant shortening of the flexor muscles, particularly in the knee joint (s750.373).

The challenge of recruiting various body segments creates a complete reliance on caregivers and assistive technology when transferring between surfaces (d420.4, e120 + 4, e310 + 4). The decline in global motor skills impacts coordination between the arm and hand, resulting in less fluid performance of movements such as pulling, pushing, reaching, or turning objects (d445.4).

As a result, they also have difficulties with activities that require intricate hand movements. However, they are more effective with supported stabilisation of the upper limbs and prior preparation by others of objects that they need to handle, placed within a short reach or in the hand itself, such as handling a console controller (d440.3, d920.1). The individual does not physically transport objects from one location to another (d430.4). To move around, he uses an electric-powered wheelchair adapted to his specific needs. Prior positioning is required to use the wheelchair (d465.1). The family home has been designed to accommodate electric wheelchairs and other necessary adaptations (e155 + 4). For longer journeys outside of the house, he relies on family transportation. The responsibility of ensuring safety systems are in place and making the journey falls on the carers (d470.2). Adapted public transport is not available in their area of residence (e5400 + 0).

The participant relies on carers for basic activities of daily living (BADLs), with the level of assistance varying (d510–520.4, d530.3, d540.4, d550–570.3, e310 + 4). Generally, the instrumental activities of daily living (IADLs) are substituted (d630–640.4, e310 + 4). The family does not benefit from the support of an external personal carer (e340 + 0).

The participant reports difficulties in maintaining informal social relationships, catalysed during the pandemic, using general communication technologies such as digital social networks to contact members of their personal network (d750.2, e1250 + 3). He maintains relationships with close family members and various extended family members (d760.0).

### 2.2. Description of the Assisted Environment

#### 2.2.1. Starting Point

**Figure 2:**
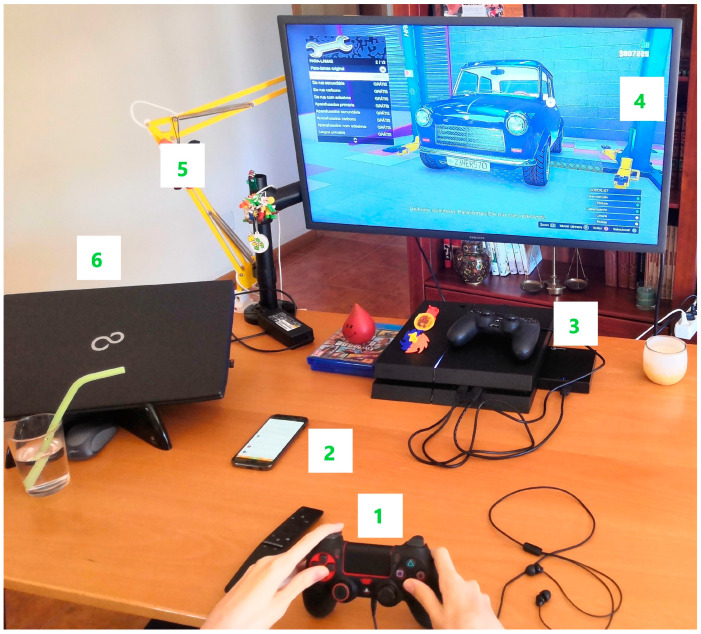
Leisure station: 1. console controller; 2. personal smartphone; 3. console; 4. television; 5. lamp.; 6. laptop.

The initial unstructured data allowed for the identification of two priority areas for adaptation based on the time spent in each room: the participant’s bedroom and the living room. The latter focused on the table where the existing devices were placed, referred to as the leisure station (see Figure 2).

It should be noted that the smartphone was often found unused in other locations due to difficulties in independent mobility. In the living room, the family also used a second television, a fan or heater, and approximately five other lighting points.

#### 2.2.2. Installed Devices

**Figure 3:**
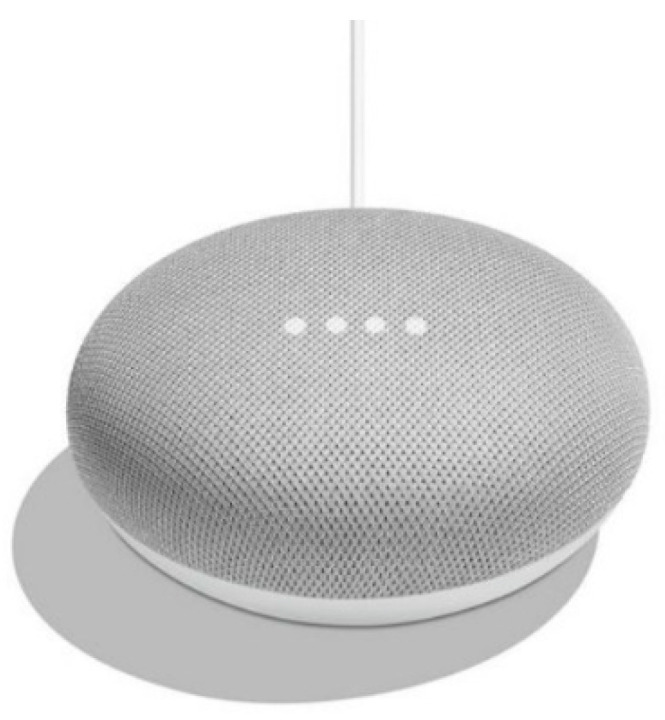
Google Nest Mini.

The decision to use SS was motivated by the participant’s mobility difficulties and the resulting constraints in reaching and activating mechanical controls. SS includes vocal and conversational interfaces that enable control of some aspects of the environment and provide opportunities for participation in new activities. The Google Nest Mini SS (Google LLC, Mountain View, CA, USA) (Figure 3) was chosen due to its balanced cost, connectivity in the required context, and understanding of the Portuguese language compared to other models. It was identified that two units were needed to cover the bedroom and living room areas.

**Figure 4:**
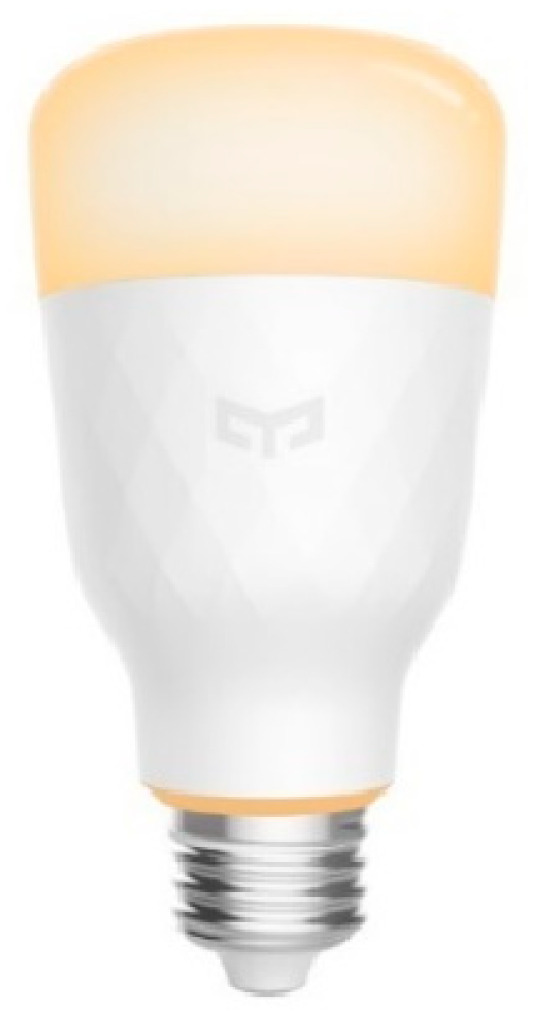
Yeelight Smart LED Bulb 1S (Dimmable) 800 Lm.

To set up the SS, the participant had to install the Google Home app (Google LLC, 2.39.1.7, Mountain View, CA, USA) on his Android smartphone and create a personal account. A Google Nest Mini unit was installed in the leisure station and connected to the Google Home app. During the process of recognising, connecting, and associating the device with the personal account, voice recognition training was performed, facilitated by the application itself. Once the SS was linked to the personal account, a smart bulb was installed in the lamp at the leisure station. The lamp chosen is the Yeelight Smart LED Bulb 1S (Dimmable) 800 Lm model (Qingdao Yeelight Information Co., Ltd, Qingdao, China) (Figure 4).

**Figure 5:**
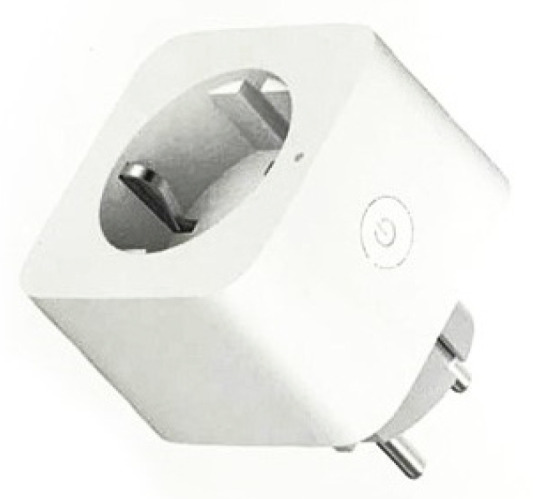
Xiaomi Mi Smart Plug (Zigbee).

A personal account had to be created on the Yeelight mobile app to identify and link the device. This association allowed S.T. to switch the light on and off or adjust its intensity from the leisure station’s lamp. After connecting the smart bulb, the next step was to connect a Xiaomi Mi Smart Plug (Zigbee) (Lumi United Technology Co., Ltd, Shenzhen, China) (Figure 5) to enable S.T. to control a temperature-controlled appliance independently. This could be a fan on hot days or a heater on colder days.

**Figure 6:**
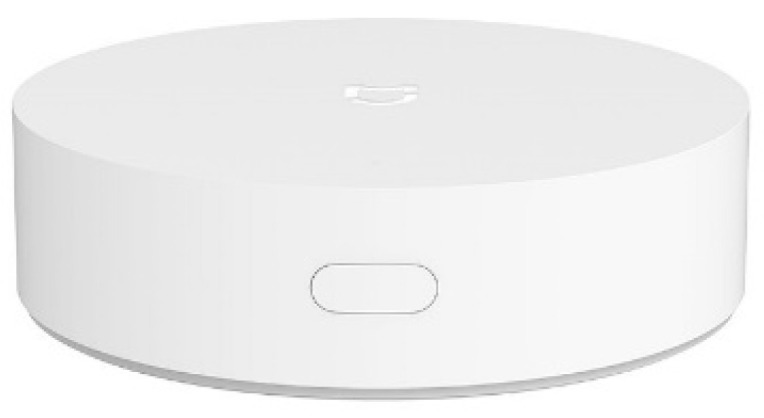
Xiaomi Hub Mi Smart Home.

The mentioned socket does not connect via WiFi but via Zigbee. This requires the installation of an intermediate home control centre that identifies and associates devices with a Zigbee connection and converts them to a regular connection. The Xiaomi Hub Mi Smart Home device (Shenzhen Lumi Lianchuang Technology Co., Ltd, Shenzhen, China) (Figure 6) was installed. It is compatible with pairing devices via WiFi, Zigbee, and Bluetooth.

The compatible application, Mi Home (Beijing Xiaomi Mobile Software Co., Ltd., 6.10.702, Beijing, China), needed to be installed to connect these devices to the participant’s smartphone. After creating an individual Mi Home account, the Hub Mi Smart Home device was associated first, which allowed for the detection and association of the Mi Smart Plug (Zigbee) socket. Once the connection to the smart plug was tested and operational, a fan was connected to it. It is important to note that the smart plug only enables the appliance to be connected and disconnected from the mains, limiting its control to on/off functionality. Therefore, the appliance must be placed in the location and intensity typically requested by S.T. to derive maximum benefit from its use.

At this stage, S.T.’s smartphone had three device control apps installed: Google Home, Yeelight (Yeelink, 3.3.08, Qingdao, China), and Mi Home. Each app was compatible with the devices of their respective brands, and all devices could be controlled via the smartphone. However, only the SS app could be controlled by voice. The Yeelight and Mi Home accounts were searched for from the Google Home App and associated with it, with the latter acting as the control centre for the associated devices. Following this connection, S.T. successfully tested voice control of the lighting and fan activation.

The process of connecting the smart socket was repeated for a second socket of the same model. During the discussion with S.T., it was determined that the most suitable connection would be to another light source located outside the leisure station, whose brightness directly affects it. S.T. could control the second lamp but only switch it on and off. This contrasts with the lamp in the leisure station, which he could manipulate the degree of luminosity because it was controlled via a smart bulb.

**Figure 7:**
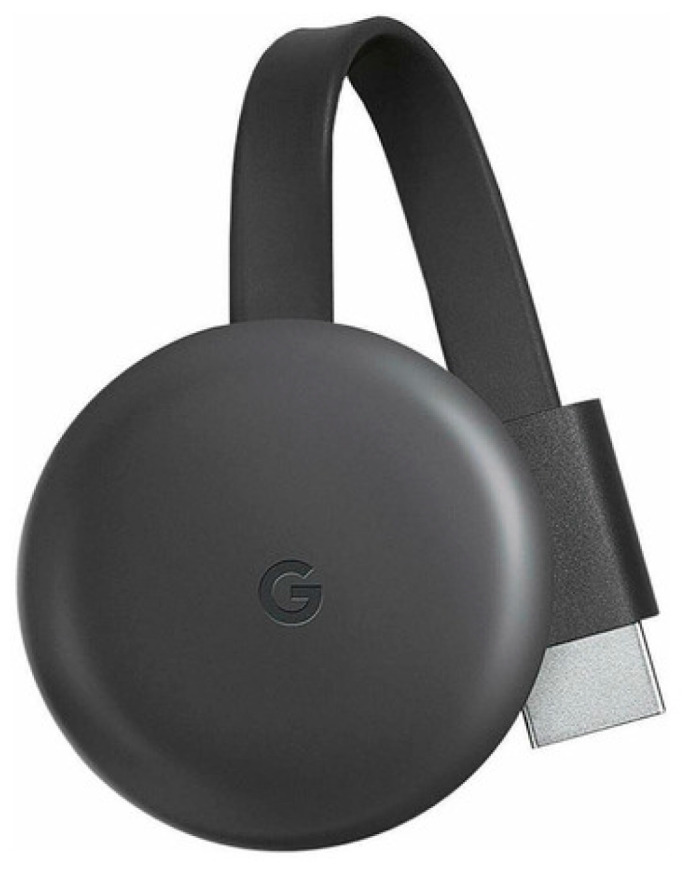
Google Chromecast.

Upon initial contact, it became apparent that the television was primarily used through the console but not necessarily to play games. It was also used for other activities, such as watching videos on YouTube and using streaming services. S.T. controlled this mode of use through the console’s controller, requiring one of the carers to provide him with the remote control. Therefore, it was deemed appropriate to connect a Google Chromecast device (Google LLC, Mountain View, CA, USA) (Figure 7) to the television. This allowed for the television to be turned on without the use of a remote control and to be used for purposes other than gaming. By linking the device to the Google Home application and streaming service accounts, S.T. was able to search for videos on YouTube or start a particular film or series on the subscribed streaming services.

No standardised techniques for SS computer control were found.

Once the devices were installed in the living room, Spotify (Spotify AB, 8.6.58.994, Stockholm, Sweden) and Google DUO (Google LLC, 107.0.334672386, Mountain View, CA, USA) accounts were created on the smartphone and linked to the Google Home app. The Spotify account enables on-demand music streaming on the SS, while the Google DUO account allows for direct calling and conversation with other DUO users through the SS without the need for a smartphone. To encourage usage, the carers were instructed to create Google DUO accounts, which they could then share with others in their close personal contacts.

**Figure 8:**
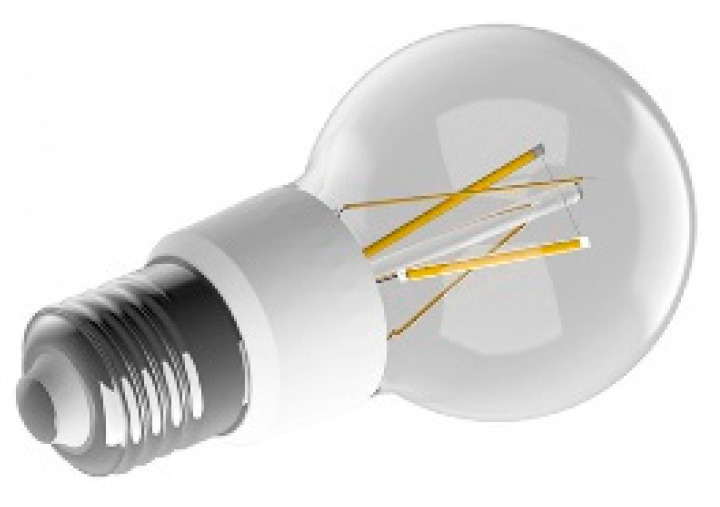
Yeelight Smart LED Filament Bulb 700 Lm.

The bedroom was adapted. The Yeelight Smart LED Filament Bulb 700 Lm (Qingdao Yeelight Information Co., Ltd, Qingdao, China) (Figure 8) was placed there, and the process of linking it to the Yeelight app was repeated, with the device automatically migrating to the Google Home app.

A second Google Nest Mini smart speaker was installed in the bedroom due to the impractical distance of the living room speaker for interaction. As S.T. uses a nasal ventilator at night, which changes the tone and projection of the voice, the speech recognition procedure for the bedroom speaker was conducted while S.T. was using it. In this room, participants were trained to set alarm clocks, reminders, and other audible warning signals to be triggered when a carer was needed. Table 1 summarises the purpose of each device and mobile application used.

**Figure 9:**
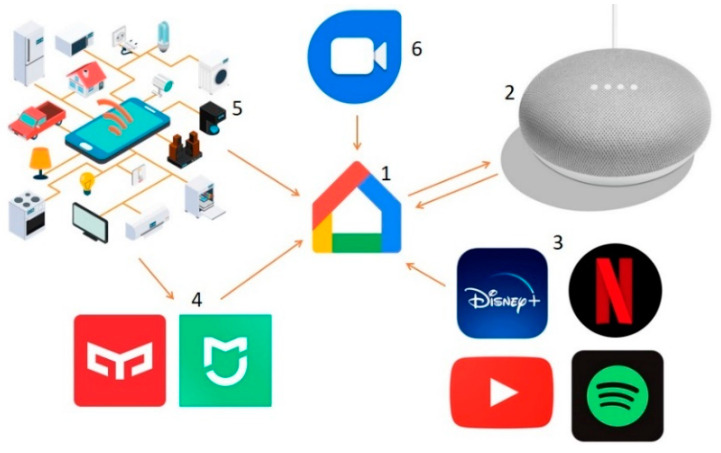
IoT connection diagram: 1. Google Home; 2. Smart Speaker; 3. Streaming services; 4. App smart devices; 5. Other smart devices; 6. Google DUO.

Figure 9 shows the scheme of IoT connections established.

### 2.3. Data Collection

Semi-structured interviews were conducted individually with the study’s participants and their carers to collect unstructured data on personal perceptions of performance, routines, environmental barriers and facilitators, and available or lacking resources, among other relevant information. These interviews were based on a pre-defined script with operational questions, validated by two specialists.

In addition, the study employed structured instruments to measure the participant’s quality of life, occupational functioning, and individual perceptions of interaction with the new devices to supplement the information and better understand the participant under study. The participant’s quality of life was measured using the abbreviated version of the World Health Organisation Quality of Life (WHOQOL-Bref) [27]. Occupational functioning was assessed using the Instrument for Screening the Model of Human Occupation (MOHOST) and the Lawton and Brody Scale to document performance in instrumental activities of daily living [28,29]. Despite being targeted to older people, the Lawton and Brody Scale encompasses domains of interest in various instrumental activities of daily living, offering valuable insights that are not available through other validated measurement tools with comparable domains. This scale can be applied to individuals within this age range or with this specific condition. Interaction with the devices in the environment was measured at the last assessment using the Portuguese version of the System Usability Scale (SUS) [30].

In order to analyse the burden, the carers also completed a structured instrument, the Questionnaire for the Assessment of Informal Carer Burden (QASCI) [31].

### 2.4. Data Analysis

The qualitative data from semi-structured interviews with the participant’s household (S.T.) and carers (mother and father) were imported, analysed, and coded for content analysis and triangulation using WebQDA 3.0 software, an online qualitative analysis tool. The corpus of data was analysed according to the principles of exclusivity between categories, intra-categorical homogeneity, completeness of the coded text, objectivity between coders, and suitability for the objectives [32]. The videotaped interviews were transcribed in full, and categorisation and coding were performed by two researchers with inter-coder agreement. The quantitative data obtained from the standardised instruments was calculated in Microsoft Excel using the associated calculation formulae for each instrument. This allowed for calculating percentage scores for WHOQOL-Bref and QASCI, percentiles for SUS, and absolute values for Lawton and Brody and MOHOST

## 3. Results

The interviews and the triangulation of information from all those involved allowed for a more in-depth understanding of the functional profile of S.T., of the household routines and resources, of the family dynamics, and of the perception and history of the disease progression, as well as the identification of expectations and needs. The subsequent topics were categorised to code the explicit and implicit references of the different participants. This allows for the exploration of individualised and more relevant perceptions with a greater number of repetitions. Table 2 shows the number of sampled units coded within each category.

Regarding the results of standardised assessments applied to S.T. (Table 3), the WHOQOL-Bref instrument shows predominantly negative variations in the “Psychological Wellbeing”, “Social Relationships”, and “Environment” domains, suggesting a gradual reduction in quality of life. However, there were no variations in general perception, and a positive variation was observed in the “Physical Wellbeing” domain.

There were no changes in the MOHOST scores across all domains, including “Motivation for occupation”, “Occupation pattern”, “Communication and interaction skills”, “Process skills”, “Motor skills”, “Environment”, and general.

This indicates a stabilisation of occupational functioning. The Lawton and Brody Scale also showed no change, with a score indicating “severe dependence” in activities of daily living.

The System Usability Scale (SUS) indicates that the introduced devices have good usability, with an overall score above the neutral point (n = 68). When applying the breakdown into domains defined by Lewis and Sauro [33], the neutral point is exceeded in the “Usability” domain, and the maximum score is achieved in “Learning”. All obtained values fall within the first percentile, resulting in an A+ score.

Regarding the application of the QASCI, there was a general trend towards an increase in burden for both carers (refer to Table 4). Both carers experienced a rise in the “Emotional burden” and “Reactions to demands” domains, while the “Financial burden” domain remained stable despite being the greatest burden. The primary caregiver experienced a decrease in the “Implications on personal life” domain and an increase in the “Mechanism of efficacy and control” and “Satisfaction with the role and with the relative” domains, which remained stable for the secondary caregiver. The most significant reduction in burden was observed in the “Family support” domain, where the caregivers reported a 12.5% difference.

## 4. Discussion

The obtained data are relevant to achieving the proposed objectives and answering the research question.

The category “Family Profile before the assisted environment” aimed to map the initial context of the direct beneficiary, S.T., and the carers. It was divided into two subcategories: “Routines” and “Family dynamics”. The category “Environment” focused on “Pre-existing devices”, “Control Mode”, and “Activities carried cut” through them. The text identifies existing and lacking resources, expectations, and needs. It also analyses performance in different occupations.

### 4.1. Routines

Family “Routines” are established based on the habits, needs, and constant presence of the individual receiving care in the household. A morning routine occurs during late hours, close to the conventional time for lunch. The caregiver prepares breakfast, organises lunch, and then attends to the individual receiving care, often waking him up around 11 am or after 11:30 am.

Generally, the primary carer wakes S.T. up—“*I get ready, in the morning, I wake up around 10 am, I go and make breakfast, I prepare lunch, and in the meantime, I come to call him, I come to pick him up. Around 11.30, sometimes more.*” (mother)—and starts hygiene care: “*…I take him to the toilet. We have a hoist from the bedroom to the bathroom that takes him up, I put him there, he does his hygiene, then I take him to his room again and I fix him up, dress him, take care of him. Then he gets up again, we put him in the chair. Then we go to the loo again because we have to brush our teeth…*” (mother). In this last point, there is a back and forth of trips to and from the bathroom, which does not contribute to the carer’s energy conservation.

After this care, S.T. goes to the leisure station, where he spends most of his time, stating that “*During the day I play Playstation, I watch videos on Youtube, I go out a bit, on Tuesdays and Thursdays I go to physiotherapy a bit*” (S.T.). It is clear from the participant’s speech that physiotherapy is the only activity he does outside his home: “*now he doesn’t like to leave the house because he’s afraid of catching the virus*” *(in an invocation of SARS-CoV-2)*” (father). The evening is spent with the family: “*we had dinner, watched a bit of telly, then I went back to playing Playstation… And then I went to bed (…) it was late, about one in the morning.*” (S.T.). The data indicate that the activities performed during active hours are primarily recreational and enjoyable, which aligns with the activity peaks observed in boys with DMD [34]. The participant’s late bedtime may explain the morning routines of both the caregiver and the participant.

Gibson et al. [35] report a pattern of difficulties in engaging in higher educational activities and work during the transition to adulthood. This may reinforce the significance of continuity in leisure activities. Lindsay et al. [6] identified school, volunteering, social, and recreational activities as the most significant occupations for young adult-aged between 19 and 28 with DMD. They found that some individuals, like S.T., do not have a defined main occupation after completing a cycle of studies. Both studies suggest that the expectation of following a normative life path (school, higher education, work) can hinder individuals from engaging in meaningful activities. Therefore, it is vital to prioritise leisure, socialising, and involvement in unpaid activities to remain active in case of unemployment [6,35].

During the night, there is a strongly established routine, motivated by the positional discomfort perceived by S.T., and his consequent need to change position in bed at least twice a night. The mother implicitly mentions the night-time uneasiness: “*I’m going to turn him, but after I turn him, it’s not long either, it’s an hour, an hour and a half.*” (mother). To this end, he calls the carers orally until they wake up, indicating the need for a continuous state of alertness on the part of the carers, who must be attentive to the faint sounds emitted by the participant due to his poor vocal projection as a result of his respiratory insufficiency.

As respiratory alterations are commonly present in the clinical condition of individuals with DMD, abnormal sleep patterns are often observed, as is the case with S.T., who requires a ventilator. The addition of physical discomfort further deviates from normative observations [36,37]. Bloetzer et al. [38] found a significant correlation between night-time immobility and sleep disturbances. S.T. does not benefit from a night-time positioning system developed for this purpose. Therefore, the family resorts to placing pillows at strategic points on the body to provide comfort, not for postural stabilisation. Sleep disturbances also affect carers, who experience reduced sleep quality due to regular interruptions, contributing to burden [39].

### 4.2. Family Dynamics

The “Family dynamics” are intuitively organised, with the mother devoting more time to caring. The father developing his professional activity, the latter testifying the following: “*Let’s say that the basis is the mother and then I help out in between. And then at night, if I need to bathe him, or put him to bed… usually I’m the one who puts him to bed*.” (father). Any variations in the mother’s routine involve liaising with the father to ensure that care is maintained: “*When I’m not around or I have to leave to go somewhere, the father is nearby and occasionally comes to see him.*” (mother). This dynamic is consistent with the demographic characterisation presented by Andreozzi et al. [40], which establishes that, in Portugal, in around 85% of families with at least one member with DMD, the mother takes on the role of main carer, and only 32.6% of carers are employed.

### 4.3. Environment

The “Pre-existing devices” are mostly concentrated in what we call the leisure station: “*I have the computer, I have the smartphone, I have the Playstation (…) the chair (…) the heater*”; “*That television I was talking about is, let’s say, I have one just to play PlayStation, it’s just connected to the PlayStation. I have another one, which is the television in the living room*” (S.T.).

The initial “Control mode” of the devices depended on motor actions and, consequently, the ordering of the controllers in functional positions; the participant even mentions, “*I can (…) if it’s within reach, it’s easy*” (S.T.), and his mother agrees that “*…we put his arms on the table for him to play*” (mother). The participant identified that the main “Activities carried out” at the leisure station consist of “*watching films on Netflix (…) and playing online games…*” (mother) and “*checking social networks…*” (S.T.), “*…watching videos on Youtube…*” (S.T.), and “*…watching the soap opera…*” (S.T.).

### 4.4. IoT Assisted Environment Implementation

“IoT assisted environment implementation” was sub-categorised into “Points for improvement”, “Advantages”, and “Experience” in relation to “Voice Assistants”, and possible “Changes in habits and routines”, “Variations in performance”, “Changes in control methods”, and “Usability” were also monitored.

Among the members of the household, only S.T. had had a momentary “experience” of interacting with voice assistants through interacting with a smartphone, without any record of previous interaction with voice assistants: “*…it was that thing of… just experimenting, for fun*” (S.T.).

After implementation, different “Variations in performance” were identified. The father says the SS has allowed S.T. to use voice commands to activate tasks traditionally carried out using a smartphone. This device is only used occasionally due to difficulties in physical access. The parent even emphasises that voice activation has become an enabler, and its use is unavoidable: “*…he certainly wouldn’t use it if it weren’t for the fact that he had this device*” (father). An example of this is making calls through the SS—“*I can call them through that device*” (S.T.)—which constitutes a “Changes in control methods” of a pre-existing device in their ecosystem. This possibility of using the SS to compensate for deficits in motor skills was also identified in Kim and Choudhury’s [41] work with elderly people. Associated with the suggestion of using SS instead of smartphones to make calls, these authors identified, among the population studied, the fear of provoking a tendency towards physical inactivity. The fear that use will lead to inactivity contrasts with the perspective obtained in the study described here: that use promotes “Autonomy”. The prospect of gaining autonomy with the use of the devices and the consequent increase in self-esteem is recognised and valued by the carer, who points out that “*we, by nature, like to be autonomous. So if he has taken steps in this direction, he obviously feels better*” (father), which suggests that the perception of benefit may be related to the degree of physical dependence. This functional change has even triggered “Changes in habits and routines” in the father, who says that when the primary carer is absent, he feels more at ease to carry out tasks in other parts of the house, as he has the security that if necessary, S.T. will call: “*what gives me is a bit more flexibility (…) because I have activities outside the house, in another area and if he didn’t have another faculty I wouldn’t be so comfortable*” (father). He adds that previously, he would have had to stay home in the same situation: “*I couldn’t be outside*” (father). The change in the carer’s habits in a specific situation is in line with the observations of Pradhan et al. [42], who found that by training people with disabilities to use the SS and control a smart home, the user’s independence increased, which is indirectly helpful for carers. Also, concerning communication, the main carer said that S.T. “*sends me a message to the living room because you know I’m in the kitchen, to the device that’s in the living room*”. (mother). This can be seen as a “Variation in performance” in the request for help from the bedroom to the kitchen, with rooms at opposite ends of the house, which previously created barriers to requests for help. The carer explains that S.T.’s strategy involves giving an instruction to the SS in the bedroom to communicate with S.T. at the leisure station, which is in the room adjacent to the kitchen, making it audible.

The ability to control the light points converted with IoT devices is a “Performance Variation” valued by all those involved, with the mother pointing out that S.T. “*…comes from anywhere and wants to go to his room and gets there ‘google, open the light*”, an aspect also identified by the father: “*He uses the lights a lot*” and by himself “*If I want to go alone and it’s dark, I can go alone and turn on the light*”. The carers also highlight autonomy in controlling environmental temperature management devices: “*now in the summer he turns on a fan, he manages that*” (father) and “*he asks to turn on the heater in the winter or the fan in the summer*” (mother). The control of lights and temperature management devices are frequently suggested in the literature and distinguished by SS users with some disability, revealing consistency with the observations made by the three participants [43,44,45,46].

Family interaction also seems to have changed with the introduction of the SS, with interaction with the devices emerging as a form of humour and a way of easing the care situation: “*he laughs a lot because I talk to Google as if it were a person, you know? And he says “Oh mum, that’s Google”. This also makes for a lighter atmosphere*” (mother) and “*Sometimes we ask the system questions, funny things and for it to tell us a joke, for example*” (father). These results are in line with those obtained in the work by Smith et al. [47], albeit with a different population. When studying the attribution of SS in people with intellectual disabilities, they identified an involvement with the devices in activities such as conversations, joke requests, and music streaming, influencing the emotional state [47]. Music streaming is highlighted by S.T., who values the possibility of listening to music in the bedroom during waiting times and at night—“*…now I can listen to music at night, without having to ask anyone*” (S.T.)—as well as carrying out autonomous Internet searches and using tools:“*when I’m curious, I ask the device and it answers. That way I don’t have to ask someone for help to search on the smartphone*” (S.T.) and “*sometimes I use the translator*” (S.T.). Also noteworthy is the independent control of basic TV commands: “*It was the fact that I could also switch on the TV I usually play games on, just by talking. Before I had to press the button on the remote and I couldn’t place my hand on the table, I had to ask someone.*” (S.T.) [44].

### 4.5. Usability

All the participants referred to “Points for improvement” related to voice assistants. The carer identifies “*…diction issues if we are too far away from the system (…) sometimes the system doesn’t understand and gives an answer that has nothing to do with it (…). The system doesn’t interpret well because the sound quality wasn’t the best because we’re too far away*”. In the same vein, S.T. mentions “*Let’s just say that sometimes the assistant is a bit stubborn (…) a person says one thing and sometimes he understands something else. Other times, one person speaks well but he doesn’t understand, then another person comes along, says the same thing and he understands*”; “*All it takes is for the person to be a bit more clogged up and he doesn’t understand very well*” and “*sometimes, out of the blue, he starts talking to himself*”, the latter reference being potentially related to the SS’s proximity to the television, and the sounds emitted could be interpreted as a command. The primary carer relays an idea from S.T.: “*He even says, “Oh mum, it’s a pity this can’t open the window (…)*”, and in this application, it is possible, through motors in the windows, but it was not included for budgetary reasons. These references signal potential barriers to understanding. In addition, these devices are not yet optimised for European Portuguese, so participants have to use Brazilian Portuguese intonation to make themselves understood by the device. However, it is worth noting that Google Assistant has the highest comprehension rate, at 95%, in optimal conditions, compared to Amazon Alexa and Apple Siri. Additionally, Google Assistant provides answers in the shortest time [45].

Despite the identification of these points for improvement, the general “Usability” considerations are positive, proving to be a system that is easy to adapt to, learn and use, as corroborated by all the interviewees: “*Yes, he adapted very quickly. Faster than me.*” (father); “*Initial instructions and a bit of practice and it’s done*” (father) and “*I think it was normal when you’re starting out with a new device. It was adaptation, I didn’t think it was anything serious*” (mother). These observations align with the scores obtained on the SUS, completed by S.T., indicating straightforward learning and usability in individual domain assessments. Masina and colleagues [46] state that the Google Nest device has good usability and is user-friendly when used by people without speech articulation deficits, like S.T.

### 4.6. Carer Burden

The burden on carers is evident, generally implicit in speech and often explicit, as well as being quantified in the QASCI (18.2% mother; 14.4% father). “Financial” burden emerges as the highest domain (50% mother; 50% father) and is a concern expressed mainly due to a perceived lack of social support or the fact that it is difficult to obtain it to buy equipment, access therapies, assign a personal assistant, among other things: “*working to pay someone wasn’t easy either, so it might as well be me. But I needed someone to replace me in certain activities so I could be a bit freer*” (mother); “*Look, physiotherapy, if we lived in a decent country, physiotherapists would certainly come to our house, I have no doubt about that*” (father), “*I thought about changing the house I had, but it wasn’t possible (…). So I have to build a new house. I’ve had absolutely no support, zero*” (father). In their analysis of the cost of illness in people with DMD in Portugal, Smith et al. [47] found that families face a high economic burden, which tends to increase as the disease progresses and peaks during the transition from the ambulatory to the non-ambulatory phase.

The area of family support also showed high values (25% mother; 50% father), even with a reduction of 12.5%. Despite the quantified decrease in the perception of the need for human help after the introduction of the devices, the idea of the need for formal support remains in the content analysis of the statements: “*…there had to be help from a third party, at least for part of the day or night*” (father). Although not directly related to this study, the literature on the Portuguese context suggests that implementing the informal carer statute approved in Portugal in 2019 could be an alternative method of reducing the perceived need for help. This could be achieved by relieving the tasks performed and providing greater sociofinancial compensation, including more financial support, care training, psychological support, and assistance in reconciling personal and professional life [48].

The QASCI results suggest that emotional burden corresponds to the domain with the most marked negative variation, to which situations of criticism and/or ambiguity can contribute, showing latent frustration on the part of the cared-for person and their carer—“*So he says I do everything wrong. And I’m like ‘I’m making a sacrifice to come here to the room…*”, “*and I’m making a sacrifice, Mum, of having to call and knowing that you’re coming here and you’re going to suffer from it*” (mother)—and thoughts about the future: “*now the question is ‘what am I going to do?*” (father). These emotional changes align with the correlations established by Pyae and Joelsson [49], who associate the stress resulting from continuous caregiving with the development of depressive symptoms. The same study indicates that caregiving stress is higher among women and in low-income contexts, a situation similar to that found in the study by Labisa and colleagues [50]. Kim [51] suggests that concerns about the future arise due to the progression of the disease and the ageing of the carer, which may affect their ability to continue providing care.

Given the aspects related to burden in the study context already discussed, the low quantification of burden related to satisfaction with the role and the family member (5% for both mother and father) was unexpected. This phenomenon, although surprising, is not isolated. Pangalila et al. [52] demonstrate that carers of individuals with DMD develop resilient personalities, which can promote psychological adaptation and role satisfaction.

## 5. Conclusions

This study identified variations in the performance of individuals with DMD and their carers, facilitated by introducing smart devices and SS as an environmental control unit. The study used qualitative data to investigate alternative voice access so that individuals could control the devices entirely. However, it is acknowledged that implementing automation, such as a light sensor detecting a decrease in light and smart bulbs gradually increasing their intensity to maintain environmental visibility, could enhance this compensatory approach. Therefore, it is recommended that future studies include this technology.

Structuring an IoT environment controlled by SS could potentially increase the participation of people with DMD and their carers. It could also be a low-cost alternative to traditional home automation systems. However, it is important to note that the simultaneous purchasing of several IoT devices can represent a significant percentage of a family’s monthly budget. Therefore, a phased purchase could be a more feasible option. The study’s limited budget restricted the range of devices that could be purchased. Additionally, the observation period was affected by S.T.’s respiratory illness, which may have impacted the final quantitative data for all participants.

It is important to pursue scientific knowledge regarding the introduction of IoT devices available on the open market, increasing the evidence.

## Figures and Tables

**Table 1:** Summary of devices and mobile applications used.

Device/App	Description/Function	Purpose	Units
Google Nest Mini	Voice-recognised interface that transmits commands to the other devices	Voice interaction with IoT networks	2
Yeelight Smart LED Bulb 1S (Dimmable)	Smart lamp switched on using WiFi with voice commands received by Google Nest	Leisure station lighting (console in living room)	1
Xiaomi Mi Smart Plug (Zigbee)	Smart socket activated via Zigbee	Control of a second light point in the room	1
Xiaomi Hub Mi Smart Home	Zigbee to WiFi signal conversion gateway	Conversion of Zigbee connections to WiFi. Allows Zigbee devices to be linked to the smartphone	1
Yeelight Smart LED Filament Bulb	Smart light bulb activated via WiFi by voice commands received by Google Nest	Bedroom Lighting	1
Google Chromecast	HDMI streaming media adapter	Enabling the television to play content and switch it on/off by alternative means	1
Yeelight App	Yeelight device recognition and connection app	Connect selected lamps to the smartphone	---
Mi Home App	Xiaomi device recognition and connection app	Enable Smart Socket and Hub connection	---
Google DUO (now Google Meet)	Voice and video calling app	Enable voice and video calls to be made and received via SS	---
Disney+, Netflix, Youtube, Spotify	Video and music streaming services	Browse video and music on-demand streaming services through the smart speaker	---
Google Home App	Managing Central control hub software for smart home devices	Link Chromecast and Nest, import Yeelight and Mi Home apps with the respective devices link Google DUO accounts and streaming services	---

**Table 2:** Content analysis and categorisation of qualitative data.

Categories	Subcategories		Sampled	Sources
Family profile before the assisted environment	Routines		31	4
	Environment	Pre-existing devices	7	2
		Control mode	4	2
		Activities carried out	13	4
	Family dynamics		13	4
	Resources		20	3
	Expectations		13	4
	Performance	Requirements for participation	9	3
		Participation	9	1
		Requests	7	1
		Difficulties	3	1
IoT-assisted environment implementation	Voice Assistants	Points for improvement	8	3
		Advantages	11	3
		Experience	3	1
	Changes in habits and routines		13	3
	Variations in performance		19	3
	Changes in control methods		12	2
	Usability		9	3
Analysis of carer burden	Perception of burden		10	3
	Supported activities		28	5
	Reduced support		4	2
	Social		2	2
	Leisure opportunities		6	2
	Financial		5	2
	Changes to life projects		4	2
	Constraints in activity		5	2
	Future		2	1
Analysis of self- and caregivers perception	Autonomy		2	2
	Independence		4	2

**Table 3:** S.T. structured assessment results.

Instrument	Domain	Initial Score	Final Score	Variation
WHOQOL-Bref	General Perception	70%	70%	0%
	Physical Wellbeing	68.6%	71.4%	2.8%
	Psychological Wellbeing	86.7%	76.7%	−10%
	Social Relationships	60%	33.3%	−26.7%
	Environment	82.5%	80%	−2.5%
MOHOST	Motivation for occupation	13	13	0
	Occupation pattern	12	12	0
	Communication and interaction skills	16	16	0
	Process skills	16	16	0
	Motor skills	8	8	0
	Environment	13	13	0
	General	78	78	0
Lawton and Brody	General		1	0
SUS	Usability		90.6	-----
	Learning		100	
	General		92.5	

**Table 4:** Results of the structured carer assessment.

Instrument	Domain	Initial Score		Final Score		Variation	
		Mother	Father	Mother	Father	Mother	Father
QASCI	Emotional Burden	18.8%	18.8%	43.8%	37.5%	25%	18.7%
	Implications for personal life	45.5%	38.6%	47.7%	38.6%	2.2%	0%
	Financial Burden	50%	50%	50%	50%	0%	0%
	Reactions to demands	10%	10%	15%	15%	5%	5%
	Efficacy and control mechanism	33.3%	33.3%	25%	33.3%	−8.3%	0%
	Family support	37.5%	62.5%	25%	50%	−12.5%	−12.5%
	Satisfaction with role and family member	10%	5%	5%	5%	−5%	0%
	General	13.6	11.4%	18.2%	14.4%	4.6%	3%

## Data Availability

The data collected have not been made available due to the possibility of the study participants being identified.

## Supplementary Materials

The following supporting information can be downloaded at: https://www.mdpi.com/article/10.3390/ijerph21060778/s1, Table S1: ICF qualifiers for body functions (b), activities and participation (d) and environmental factors (e)., Table S2: ICF qualifiers for structures (s).
