# Supplementary material for: Smart Speakers as an Environmental Control Unit for Severe Motor Dependence: The Case of a Young Adult with Duchenne Muscular Dystrophy
Source: Int J Environ Res Public Health. 2024 Jun 14;21(6):778. doi: 10.3390/ijerph21060778 (PMC11204232; doi:10.3390/ijerph21060778)
Supplement: Supplementary file 1 [file ijerph-21-00778-s001.zip › ijerph-2999375-supplementary.pdf]

Table S1. ICF qualifiers for body functions (b), activities and participation (d) and environmental factors (e).

| | |
|---|---|
| In the ICF, body functions (b) and activities and participation (d) are categorised as follows:<br>xxx.0 NO problem (none, absent, insignificant) 0–4%<br>xxx.1 MILD problem (slight, small, …) 5–24%<br>xxx.2 MODERATE problem (medium, regular, …) 25–49%<br>xxx.3 SEVERE problem (large, extreme, …) 50–95%<br>xxx.4 COMPLETE problem (total, …) 96–100%<br>xxx.8 not specified<br>xxx.9 not applicable | In the ICF, environmental factors (e) are categorised as follows:<br>xxx.0 NO barrier (none, absent, scarce…) 0–4%<br>xxx.1 MILD barrier (slight, weak…) 5–24%<br>xxx.2 MODERATE obstacle (medium, fair…) 25–49%<br>xxx.3 SEVERE barrier (high, extreme…) 50–95%<br>xxx.4 COMPLETE barrier (total…) 96–100%<br>xxx + 0 NO facilitator (none, absent, scarce…) 0–4%<br>xxx + 1 MILD facilitator (slight, weak…) 5–24%<br>xxx + 2 MODERATE facilitator (average, fair…) 25–49%<br>xxx + 3 SUBSTANTIAL facilitator (high, extreme…) 50–95%<br>xxx + 4 COMPLETE facilitator (total…) 86–100%<br>xxx.8 Barrier not specified<br>xxx + 8 Facilitator not specified<br>xxx.9 not applicable |

Table S2. ICF qualifiers for structures (s).

| | | |
|---|---|---|
| In the ICF, the structures (s) are qualified as follows:<br>First qualifier Common qualifier with a negative scale used to indicate the extent or magnitude of an impairment: xxx.0 NO impairment (none, absent, scarce…) 0–4%<br>xxx.1 MILD disability (slight, small, …) 5–24%<br>xxx.2 MODERATE disability (average, fair…) 25–49%<br>xxx.3 SEVERE disability (large, extreme…) 50–95%<br>xxx.4 COMPLETE disability (total…) 96–100%<br>xxx.8 not specified<br>xxx.9 not applicable | Second qualifier Used to indicate the nature of the change in the corresponding body structure:<br>0 no change in structure<br>1 total absence<br>2 partial absence<br>3 additional part<br>4 aberrant dimensions<br>5 discontinuity<br>6 deviated position<br>7 qualitative changes in structure, including accumulation of fluid<br>8 not specified<br>9 not applicable | Third qualifier (suggested) Under development to indicate location:<br>0 more than one region<br>1 right<br>2 left<br>3 both sides<br>4 front<br>5 back<br>6 proximal<br>7 distal<br>8 not specified<br>9 not applicable |
